# The Increased Abundance of Commensal Microbes Decreases *Drosophila melanogaster* Lifespan through an Age-Related Intestinal Barrier Dysfunction

**DOI:** 10.3390/insects13020219

**Published:** 2022-02-21

**Authors:** Hye-Yeon Lee, Shin-Hae Lee, Kyung-Jin Min

**Affiliations:** Department of Biological Sciences, Inha University, Incheon 22212, Korea; lee.hy@inha.ac.kr (H.-Y.L.); lmjinee@gmail.com (S.-H.L.)

**Keywords:** commensal microbes, intestinal barrier dysfunction, *Drosophila*

## Abstract

**Simple Summary:**

Commensal microbes affects the host’s health, physiology, dysbiosis, and the disruption of microbiota homeostasis, which can lead to a wide range of diseases, including inflammatory bowel disease (IBD). *Drosophila melanogaster* was recently introduced as a model for human intestinal infection and pathology. Here, we show that the lifespan of flies is tightly related with the gut permeability of flies, confirming a causal relationship between gut dysbiosis and host lifespan.

**Abstract:**

Background: Commensal microbiota live in their host with a symbiotic relationship that affects the host’s health and physiology. Many studies showed that microbial load and composition were changed by aging and observed that increasing the abundance and changing the composition of commensal microbes had detrimental effects on host lifespan. We hypothesized that dysbiosis of the intestinal microbiota leads to systemic effects in aging flies as a result of the increased intestinal permeability. Methods: We used the fruit fly, *Drosophila melanogaster*, laboratory strains *w^1118^*, as a model system with many advantages for microbe–host studies. Results: The incidence of intestinal dysfunction was increased with age, and intestinal dysfunction increased the permeability of the fly intestine to resident microbes. The lifespan of flies with an intestinal barrier dysfunction was increased by removal of the microbes. Interestingly, some bacteria were also found in the hemolymph of flies with intestinal barrier dysfunction. Conclusion: Our findings suggest the possibility that, as the host ages, there is an increase in intestinal permeability, which leads to an increased intestinal microbial load and a reduction in the host lifespan. Our data therefore indicate a connection between commensal microbes and host lifespan.

## 1. Introduction

Commensal microbiota live in their host with a symbiotic relationship that affects the host’s health and physiology. In recent years, there has been active research on the treatment and prevention of diseases through changes in the commensal microbiota; furthermore, the possibility of realizing a better quality of life through these studies was suggested. Many studies have already revealed that commensal bacteria affect many physiological activities of the host, and especially immune system and intestinal health are closely related to commensal bacteria [1,2].

In general, the immune response of the host eliminates the pathogens that enter the body through ingestion of food or through other routes, such as infection. However, when the immune system is destroyed, inflammatory reactions and intestinal microbial dysbiosis are observed. Studies of intestinal microbial composition in patients with inflammatory bowel disease (IBD) showed that these patients differed from healthy adults in their microbial composition [3,4]. For example, the ileum of patients with Crohn’s disease, one of the IBDs, has shown reduced number of Firmicutes, which are known to produce anti-inflammatory agents [5]. In addition, studies in rodents have found that certain combinations of intestinal microbiota are highly effective in inducing enteritis [6]. This suggests that IBD can be treated through changes in microbial composition in the intestine, although it is not yet clear whether abnormal intestinal microbiota is causative of IBD.

Intestinal dysfunction is also closely related to the lifespan of organisms. Clark et al. and Guo et al. extended the lifespan of *Drosophila* by preventing dysbiosis-related intestinal barrier dysfunction and preventing dysbiosis through the activation of peptidoglycan recognition protein SC2 (PGRP-SC2), a negative regulator of the IMD/Rel pathway [7,8]. In addition, Li et al. prevented age-related dysbiosis through inhibition of the gut compartmentalization defect, extending the lifespan of *Drosophila* [9]. In our previous study, we demonstrated that the removal of commensal bacteria without harmful side effects increased fly lifespan, and that bacterial load was a significant determinant of lifespan [10], although the studies on the effect of commensal microbes on the lifespan of *Drosophila melanogaster* are controversial [7,11,12]. However, in previous studies, we did not present how increased commensal bacteria shorten the lifespan of fruit flies. Therefore, we hypothesized that the relationship between intestinal dysfunction and commensal bacterial abundance would determine the lifespan of the host.

This study focused on the relationship between commensal microbiota and host longevity in *D. melanogaster*, which is a well-established model organism in aging and host-microbe studies. Especially, the present study explores the mechanisms by which commensal bacterial load affects the host lifespan in *D. melanogaster*.

## 2. Materials and Methods

### 2.1. Fly Husbandry and Generation of Axenic D. melanogaster

Experiments were conducted using the *D. melanogaster* wild-type strain *w^1118^*, which was provided by the Bloomington Drosophila Stock Center (Indiana University, Indianapolis, IN, USA) and has been adapting to our laboratory environment over the last 10 years. The flies were cultured and reared at 25 °C and 65% humidity in a 12:12 h light:dark cycle. The sterile standard cornmeal-sugar-yeast (CSY) medium (5.2% cornmeal, 11% sugar, 2.5% instant yeast, 0.5% propionic acid, 0.04% methyl-4-hydroxybenzoate (Sigma-Aldrich, St. Louis, MO, USA), and 1% agar) was used during culture and rearing of the flies. For the sterile CSY diet, the above-mentioned CSY medium was autoclaved at 120 °C for 20 min, and all vials for food were exposed to UV light for 20 min on a clean bench.

Axenic (Ax) flies were generated by bleaching the embryos. Embryos were collected for 12 h and were dechorionated for 50 sec in 5% sodium hypochlorite solution (Wako, Japan), rinsed for 50 s in 70% ethanol, and washed for 1 min in sterile distilled water [10]. Sterile embryos were transferred into sterile CSY food bottles on a clean bench. The eggs in an Ax condition were passed through repeated generations and became third-generation flies. In this study, we used the third-generation Ax fly from bleached eggs. All Ax flies were maintained on a clean bench and transferred to fresh food every two days. Axenic conditions were confirmed by plating fly homogenates on plate count agar (PCA) (A Neogen Corporation, Lansing, MI, USA) containing 0.5% tryptone, 0.25% yeast extract, 0.1% glucose, and 1.5% bacto agar, and the DNA extracted from whole-fly homogenate underwent 16S rRNA gene PCR using a bacterial 16S rRNA universal primer (27F and 1492R) provided by Macrogen (Seoul, Korea).

### 2.2. Lifespan of Smurf Fly Measurement

Newly eclosed *w^1118^* adult female flies were collected for 2 days and were reared in sterile CSY medium until the emergence of the Smurf fly. Dyed medium was prepared using CSY medium supplemented with blue dye no. 1 (2.5% *w*/*v*). Flies were transferred onto the blue-dye-containing food after starvation for 4 h and were then maintained on the dyed medium for 9 h. Because the number of Smurf flies was not sufficient for experimentation, 10-day-old flies were pre-treated with 1% dextran sodium sulfate (DSS). The DSS solution containing 5% sucrose was sprinkled on each vial containing two filter papers, and the filter papers were moderately dried for about 10–30 min. For the treatment of flies with DSS, 10-day-old flies were transferred into the prepared DSS-containing vial and incubated for 10 days. A fly was collected as a Smurf fly when the dye coloration could be observed in the body. For their survival, Smurf flies were assigned to sterile CSY food vial. Dead flies were counted every 3 h, and the number was recorded.

### 2.3. Hemolymph Collection

To make a hemolymph collection tube, a 0.5 mL centrifuge tube with a perforated bottom was placed inside 1.5 mL centrifuge tube. The thoraxes from 10 female flies were washed with 70% EtOH to remove any external bacteria, after which the thoraxes were pricked using a ϕ25 needle. To collect the hemolymph, the flies were transferred into hemolymph collection tube and centrifuged at 5000 rpm for 5 min at 25 °C.

### 2.4. Bacteria Culture Conditions

*Lactobacillus* was grown on 5.5% MRS media (*Lactobacilli* MRS Broth, BD & Difco, Sparks, MD, USA) containing 1% peptone, 1% beef extract, 0.5% yeast extract, 2% dextrose, 0.1% polysorbate, 0.2% ammonium citrate, 0.5% sodium acetate, 0.01% magnesium sulfate, 0.005% manganese sulfate, 0.2% dipotassium phosphate, and 1.5% bacto agar (BD & Difco). *Acetobacter* was grown on *Acetobacter*-selective (AS) media containing 2.5% D-mannitol (BD & Difco), 0.5% yeast extract (BD & Difco), 0.3% peptone (BD & Difco), and 1.5% bacto agar. All microbes were incubated at 29 °C.

### 2.5. Quantitative Analysis and Identification of Bacteria

For CFU determination, dissected guts from 6 females were homogenized and plated onto MRS media or AS media. At least 3 replicates were established for each group. For microbe isolation, hemolymph from 10- or 50-day-old (non-Smurf or Smurf) flies were plated on a MRS and AS media plate. After incubation of a single colony at 29 °C for 3 days, each colony was transferred to MRS media broth or AS media broth. After culture for 24 h, the cell walls of isolated microbes were broken down by bead beating using Glass Beads 0.1 mm in diameter (BioSpec Products, Bartlesville, OK, USA). PCR assays were performed with a 55 °C annealing temperature and 45 cycles with the universal primers 27F and 1492R. PCR products were sequenced by using 16S sequencing (Macrogen Inc., Seoul, Korea) with the universal primers, 518F and 800R, and then analyzed by using EzTaxon blast and NCBI blast.

### 2.6. Fluorescence Imaging

Differentiation of intestinal stem cells caused by intestinal epithelial damage was observed by fluorescence image of the escargot (esg), expressed in intestinal stem cells and enteroblasts of the fly midgut. Newly eclosed esg-GPF adult female flies were collected for two days and were fed 0%, 0.5%, 1%, or 5% dextran sodium sulfate with 5% sucrose for 10 days. The guts were dissected in PBST (phosphate-buffered saline + 0.1% Triton X-100) solution. The esg-GFP fusion protein was observed using an Olympus IX71 inverted microscope equipped with a U-RFL-T (Olympus) mercury lamp and a TH4-200 (Olympus) photosystem.

### 2.7. Reverse Treanscriptase-Quantitative PCR

**Figure 1:**
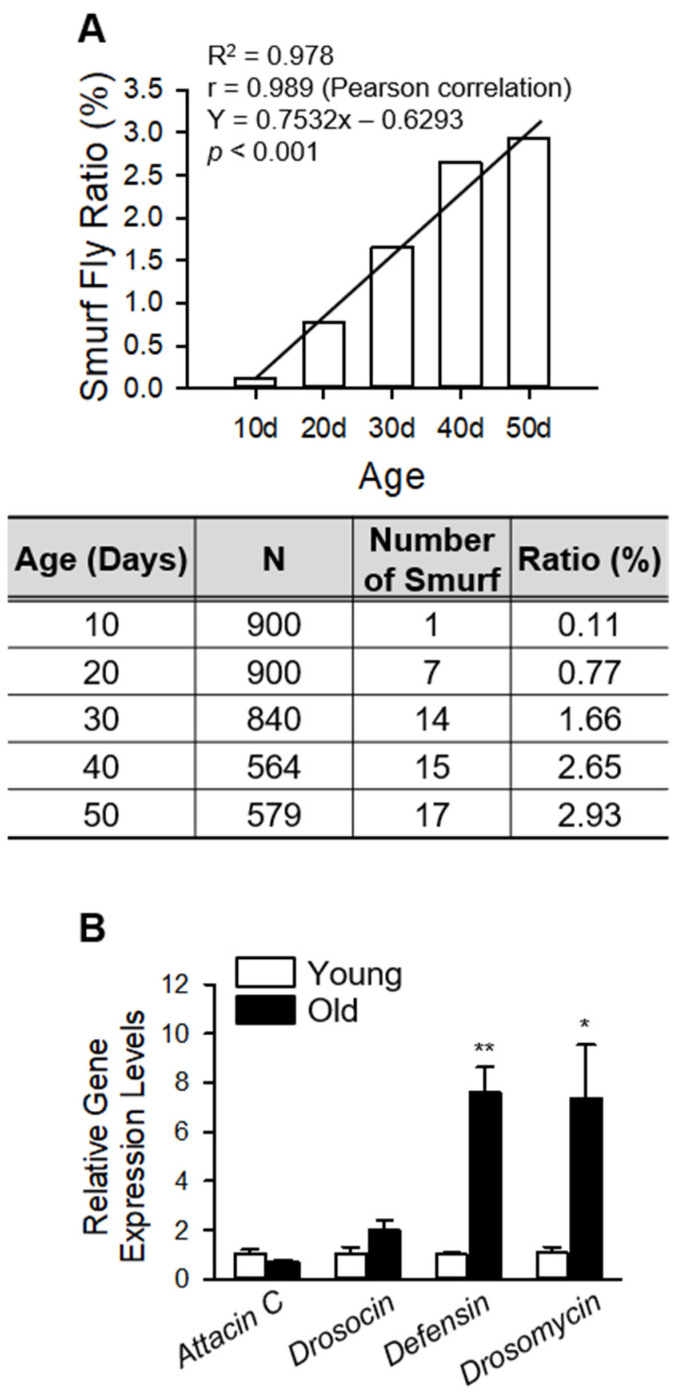
Aging increases the incidence of intestinal dysfunction and the expression of AMPs in the fly. (**A**) Smurf fly incidence is significantly increased with age. (**B**) The mRNA levels of attacin C, drosocin, defensin, and drosomycin were analyzed in the whole bodies of 10-day-old (young) or 50-day-old (old) flies. Asterisks indicate significant differences between young and old flies for each gene (*t*-test, * *p* < 0.05, ** *p* < 0.005).

**Figure 2:**
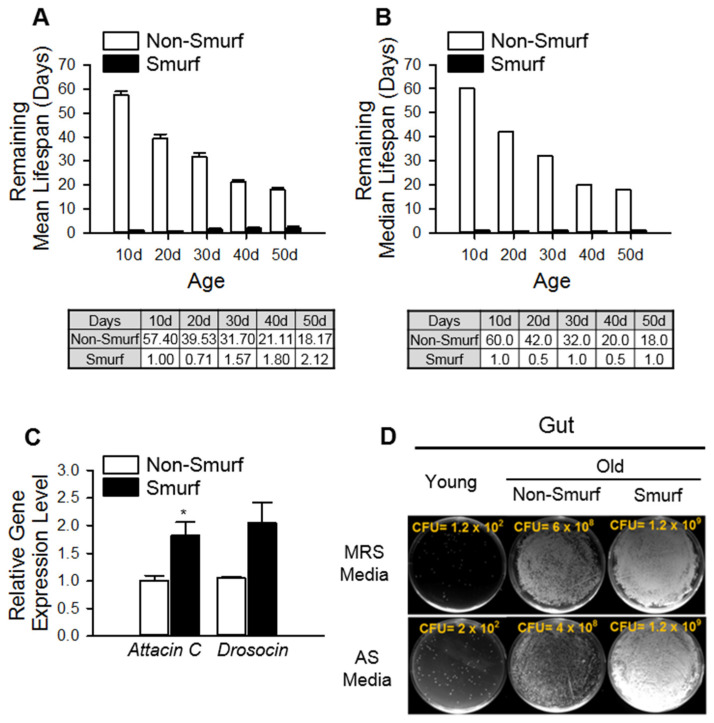
Flies with an intestinal barrier dysfunction have a severely decreased remaining lifespan regardless of their age, and also show increased expression of AMP genes and the presence of microbes in the hemolymph. (**A**,**B**) The remaining mean (**A**) and median (**B**) lifespans of non-Smurf or Smurf flies. Smurf flies have < 2.2 days remaining lifespan regardless of their chronological age. (**C**) The mRNA levels of attacin C and drosocin were analyzed in the 50 guts of 30-day-old non-Smurf and Smurf flies. Asterisks indicate significant differences compared to young flies for each gene (*t*-test, * *p* < 0.05). (**D**) The total number of CFUs from 10-day-old, 50-day-old non-Smurf, and 50-day-old Smurf flies in *Lactobacillus*-selective (MRS) media (upper) or *Acetobacter*-selective (AS) media (lower) plates.

The total RNA was extracted from 10- or 50-day-old 15 female flies for Figure 1B, and from 5 non-Smurf or Smurf female flies using RNAiso (Takara Bio, Kusatsu, Japan), as shown in Figure 2C. The total RNA (2 μg) was reverse transcribed using M-MLV reverse transcriptase (Promega, Madison, WI, USA). RT-qPCR was performed using the Prism 7500 Sequence Detection System (Applied Biosystems, Foster City, CA, USA) and TOPrealTM qPCR 2 × PreMix (Enzynomics, Daejeon, Korea) according to the manufacturer’s instructions. At least three replicates were established for each group, and all experiments were repeated at least three times. Relative expression levels of the target genes were analyzed using 2^∆∆Ct^. The data are presented as the mean ± standard error of the mean. Ribosomal protein 49 (rp49), the stable housekeeping gene, was used as the internal control. rp49, Forward (F): 5′-ATC GGT TAC GGA TCG AAC AA-3′, Reverse (R): 5′-GAC AAT CTC CTT GCG CTT CT-3′; Attacin C (F): 5′-CCA ATG GCT TCA AGT TCG AT-3′, (R): 5′-AGG GTC CAC TTG TCC ACT TG-3′; Drosocin, (F): 5′-ATT TGT CCA CCA CTC CAA GC-3′, (R): 5′- GGC AGC TTG AGT CAG GTG AT-3′; Defensin, (F): 5′-GTG GAT CCA ATT CCA GAG GA-3′, (R): 5′- CAC AGA GCG AAA CGA AAT CA-3′; Drosomycin, (F): 5′-AGC GCG GAT GGA ACG ATA TT-3′, (R): 5′-CAC AAT GCC CAC GCT CTT GT-3′.

### 2.8. Statistical Analysis

Log-rank tests were carried out to determine the statistical significance of the results of the survival analysis. The JMP statistical package (SAS, Cary, NC, USA) was used for the analyses. The statistical probability of CFU and OTU numbers were determined by using the two-sample *t*-test. Spearman’s correlation coefficients were derived by using R 3.5.1 software.

## 3. Results

Many studies showed that the commensal bacterial abundances were increased, and intestinal dysfunction was developed as aging progresses [10,13]. Additionally, the activation of the peptidoglycan recognition protein SC2 (PGRP-SC2), plays a vital role in innate immune response, prevents dysbiosis and dysbiosis-related intestinal barrier dysfunction, inducing an extension in the lifespan of *D. melanogaster* [7,8]. Based on these studies, we hypothesized that intestinal barrier dysfunction allows for the leaking of intestinal microbes into the body cavity from the gut and leads to excessive systemic inflammation in the host, causing a reduction in lifespan. We first investigated whether the incidence of the intestinal barrier dysfunction (IBD) and immune response increased with age in the fly *w^1118^* strain that we were investigating (Figure 1). The ratio of Smurf flies, which is an indicator of flies with IBD, as described in a previous study [13], gradually increased with age (Figure 1A). The expression levels of immune-response genes in either 10-day-old (young) or 50-day-old (old) flies was measured by RT-qPCR using gene primers for three antimicrobial peptides (AMPs) (attacin C, drosocin, defensin, and drosomycin). The gene expression level of attacin C was not changed, but that of drosocin, defensin and drosomycin were increased (Figure 1B) in old flies indicating that the flies have an exacerbating immune response as they age.

Additionally, the remaining mean and median lifespans of the Smurf flies were less than 2.2 days, regardless of their chronological age (Figure 2A,B), indicating that the appearance of the Smurf phenotype is an index of immediate death. Interestingly, the Smurf flies showed increased expression levels of AMPs and microbe colonization compared with those in non-Smurf flies (Figure 2C,D), indicating that intestinal barrier dysfunction is associated with increased inflammation, which is caused by an increase in microbial abundance. Thus, flies with an intestinal barrier dysfunction have a severely decreased remaining lifespan regardless of their age, and also show increased expression of AMP genes.

**Figure 3:**
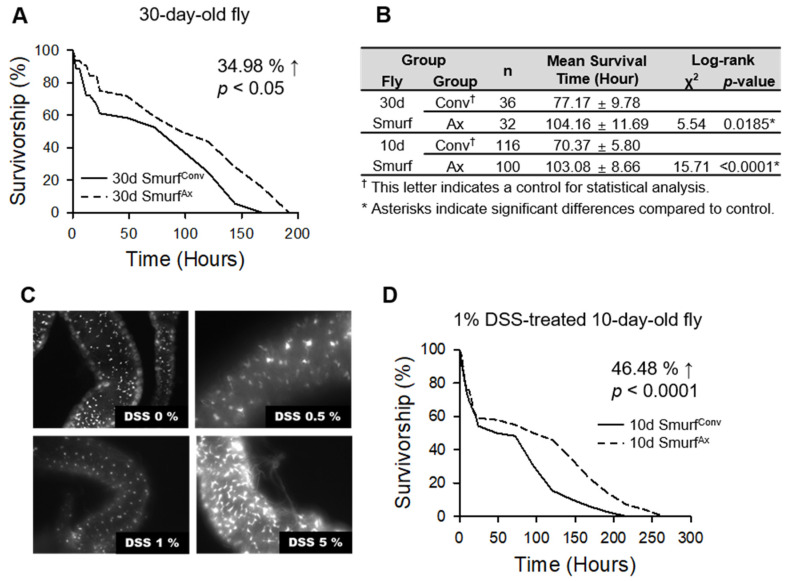
The increased mortality of Smurf flies is related to the presence of commensal bacteria. (**A**) Removal of microbes significantly increases the survival of 30-day-old Smurf flies. (**B**) Survival time of Smurf^Conv^ and Smurf^Ax^ fly. (**C**) The expression in progenitor cells of esg-Gal4 as shown by UAS-GFP fluorescence. Intestinal stem cell proliferation is induced by treatment with 5% dextran sodium sulfate, but not with 0.5% or 1% dextran sodium sulfate. These tests were performed twice with >7 guts per group. (**D**) Removal of microbes significantly increases the survival of 1% DSS-treated 10-day-old Smurf flies. The solid line indicates the survival conventional flies, and the dashed line indicates the survival of the axenic flies.

To investigate whether the increased mortality of the Smurf flies was related to commensal microbes, we measured the lifespan of Smurf flies with and without commensal microbes (Figure 3). In order to collect sufficient Smurf flies, Ax and conventionally reared (Conv) flies were aged for 30 days because the incidence ratio is low in young flies (Figure 3A). As previously reported [7], 30-day-old Ax Smurf flies lived longer than Conv Smurf flies (Figure 3A,B 30d Smurf^Conv^, 77.17 ± 9.78 h; 30d Smurf^Ax^, 104.16 ± 11.69 h, 34.98% increase, log-rank test, χ^2^ = 5.54, *p* < 0.05). To remove the effects of a decrease in other physiological parameters in the Smurf flies, we also confirmed our data with young flies. To increase the incidence ratio of the Smurf phenotype in young flies, we treated 10-day-old flies with dextran sodium sulfate (DSS), which is known to induce intestinal epithelial damage [14]. Because the differentiation of intestinal stem cells (ISCs) caused by intestinal epithelial damage affects fly lifespan, we first investigated the concentration of DSS, when DSS did not accompany ISC differentiation in esg-GFP flies (Figure 3C). Interestingly, the data showed that ISC differentiation was induced by 5% DSS treatment, but not by 0.5% and 1% DSS treatment. However, 1% DSS treatment decreased the lifespan of flies, but 0.5% DSS treatment did not affect the lifespan of flies, indicating intestinal dysfunction related with lifespan occurred only in 1% DSS (data not shown). Because 1% DSS treatment affects the lifespan of flies without ISC differentiation, we used 1% DSS in our following experiment. Next, we collected 10-day-old Smurf flies (Ax and Conv) that had been treated with 1% DSS for 1 week and measured the lifespan of the Smurf flies. Similarly, survival increased in the 10-day-old Ax Smurf flies (10d Smurf^Ax^, 103.08 ± 8.66 h) compared to the 10-day-old Conv Smurf flies (10d Smurf^Conv^, 70.37 ± 5.80, 31.73% decrease, log-rank test, χ^2^ = 15.71, *p* < 0.0001) (Figure 3B,D), indicating that the shortened lifespan of the Smurf flies was at least partly related to commensal microbes.

**Figure 4:**
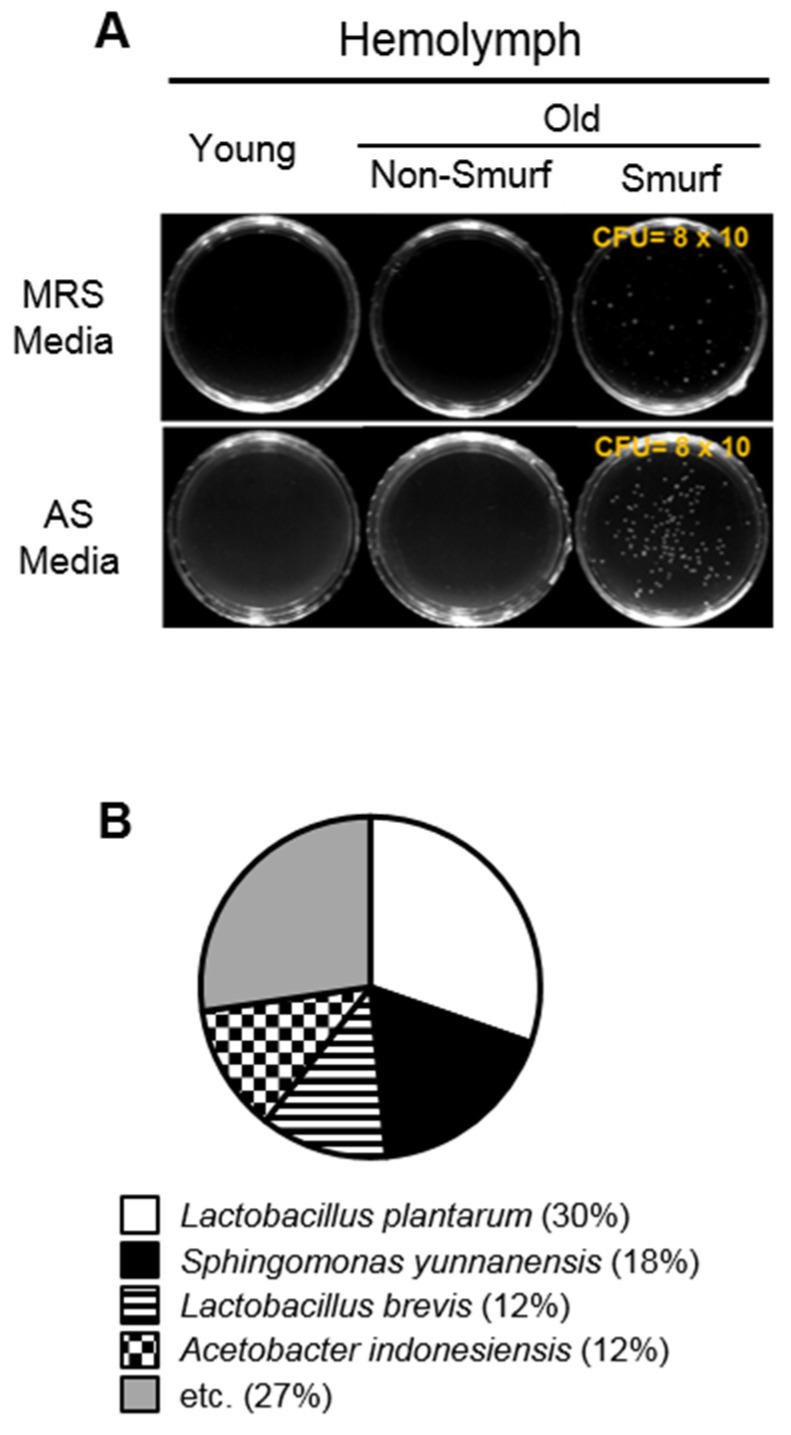
Intestinal dysfunction increases the permeability of microbe into the body cavity. (**A**) Colony-forming units on *Lactobacillus*-selective (MRS) media (upper) or *Acetobacter*-selective (AS) media (lower) plates are found only in hemolymph of Smurf fly, not in the hemolymph of non-Smurf flies (**B**) as well as 1% DSS-treated 10-day-old Smurf flies. (**B**) Pie chart showing the bacterial composition found in the hemolymph from Smurf flies. The chart shows the major species detected by sequencing of 16S rRNA genes.

To determine whether the intestinal microbes leak into the body cavity of flies through the loosened intestinal barrier, we collected hemolymph from young, old non-Smurf, and old Smurf flies, and measured the number of CFUs in hemolymph (Figure 4A). As expected, colonies were only found in the hemolymph of old flies, but not in that of young flies. Moreover, the number of CFUs was increased in Smurf flies compared to that in non-Smurf flies, although, interestingly, the flies were of the same age. We identified the different bacterial species found in the colony from Smurf fly hemolymph (Figure 4B). *Lactobacillus plantarum* (30%) was the most dominant bacterial species in the hemolymph from Smurf flies and followed by *Sphingomonas yunnanensis* (18%), *Lactobacillus brevis* (12%) and *Acetobacter indonesiensis* (12%). Taken together, these data suggest that intestinal barrier dysfunction allows the commensal microbes to enter into the body cavity of flies, and this permeation ultimately causes an increase in fly mortality through increased systemic inflammation.

## 4. Discussion

The mechanism by which commensal bacteria can regulate host lifespan is a fascinating topic. Our data indicate that increased commensal microbial abundance might reduce the host lifespan through the chronic inflammation initiated by the intestinal barrier dysfunction with aging. Interestingly, the mean survival time of Smurf^Ax^ flies was not different in 10-day-old and 30-day-old flies, whereas the mean survival time of 30-day-old Smurf^Conv^ flies was longer than that of 10-day-old Smurf^Conv^ flies (Figure 3), indicating that commensal bacteria in 10-day-old flies may have a more adverse effect on host lifespan than those in 30-day-old flies.

Some studies have shown that preventing dysbiosis by inhibiting IBD increases the host lifespan [7,8], indicating that the effect of dysbiosis on the host lifespan is closely related to intestinal barrier dysfunction. However, in our study, the removal of the commensal microbe did not completely recover the lifespan of the Smurf fly (Figure 3). This result implies that the short lifespan of Smurf flies cannot only be explained by the chronic inflammation caused by intestinal barrier dysfunction but flies with an intestinal barrier dysfunction must also have other physiological defects. One limitation of our study is that we did not confirm whether the bacteria found in the hemolymph from Smurf flies originated in the intestine. In future studies, it will be important to confirm that bacteria fed to old flies can indeed leak into the body cavity from the gut.

As mentioned above, there is growing evidence indicating that commensal microbes, directly and indirectly, affect the lifespan of a host. However, the studies on the effect of commensal microbes on the lifespan of *D. melanogaster* are controversial. These differing results could be due to differences in the composition of commensal microbiota of flies maintained in the laboratory. For example, the mono-association of flies with *Gluconobacter morbifer*, which is observed in certain laboratories, was reported to reduce the lifespan of these flies [15]. In addition, other bacteria such as *Weissella paramesenteroides* [16], *Corynebacterium variabile* [17], *Commensalibacter*, and *Serratia* [18] are reported specifically in different laboratories. In our previous study, we showed that *Acetobacter persici* and *L. brevis* were dominant in the gut of young flies, while *Acetobacter malorum* and *L. plantarum* were dominant in the gut of old flies in our laboratory fruit flies [10]. However, in hemolymph, the composition of the predominant bacteria was altered (Figure 4B) indicating that the affinity of leaking into the hemolymph is different for each bacteria species. Thus, bacterial species leaking into hemolymph through intestinal barrier dysfunction can become more diverse as fruit flies have a different composition of commensal bacteria in each environment. On the other hand, it was observed that the ratio of *L. plantarum* was very dominant in the hemolymph in Figure 4B, and it can be inferred that more bacteria were leaked because they are the dominant species in the gut of the aged fruit flies.

## 5. Conclusions

Taken together, our findings suggest that as the host ages, increased intestinal permeability may have led to the migration of commensal bacteria from the gut to the hemolymph, resulting in increased systemic inflammatory responses in fruit flies, possibly reducing lifespan. The present study indicates that a decreased lifespan by intestinal dysfunction is directly related with the migration of commensal bacteria in the host body.
